# Atypically Shaped Setae in Gall Mites (Acariformes, Eriophyoidea) and Mitogenomics of the Genus *Leipothrix* Keifer (Eriophyidae)

**DOI:** 10.3390/insects14090759

**Published:** 2023-09-12

**Authors:** Philipp E. Chetverikov, Samuel J. Bolton, Charnie Craemer, Vladimir D. Gankevich, Anna S. Zhuk

**Affiliations:** 1 Zoological Institute of Russian Academy of Sciences, Universitetskaya Naberezhnaya 1, 199034 St. Petersburg, Russia; vd.gankevich@gmail.com; 2 Department of Invertebrate Zoology, St. Petersburg State University, Universitetskaya Naberezhnaya 7/9, 199034 St. Petersburg, Russia; 3 Florida State Collection of Arthropods, Division of Plant Industry, Florida Department of Agriculture and Consumer Services, Gainesville, FL 32608, USA; samuel.bolton77@gmail.com; 4 Landcare Research, 231 Morrin Road, Auckland 1072, New Zealand; charniecc@gmail.com; 5 Institute of Applied Computer Science, ITMO University, 197101 St. Petersburg, Russia; ania.zhuk@gmail.com

**Keywords:** eriophyoid mites, nematalycids, mitochondrial genome, bifurcated setae

## Abstract

**Simple Summary:**

Eriophyoidea (gall mites) are a megadiverse lineage of worm-like mites that feed on vascular plants. The setae of these mites are sometimes distinctive because of their atypical shape, either bifurcated, angled or swollen, but never many-branched. Our study of eriophyoid setae revealed that bifurcated and angled setae are widely distributed across Eriophyoidea. The group of worm-like soil mites (Nematalycidae) with which they are affiliated have bifurcated and trifurcated setae. The plesiomorphic and also most common state of all mites is hyper-furcating setae (more than three branches), which are almost always represented in rows (seri-furcating). The likely explanation for the filiform and unbranched setae of Eriophyoidea is the gradual loss of branches, one by one, with the bifurcated state that is shared with Nematalycidae being an ancestral state. Angled setae are an intermediary state because they probably represent a bifurcating seta with only a single branch; the other one is completely diminished. Accordingly, hypo-furcating setae (three or fewer branches) are a synapomorphy that unites Eriophyoidea with Nematalycidae. Our phylogenetic analyses also showed that *Leipothrix*, the largest genus with a bifurcated seta on the palps, is monophyletic once *Cereusacarus juniperensis* is excluded and five species of *Epitrimerus* have been transferred into this genus.

**Abstract:**

The setae in Eriophyoidea are filiform, slightly bent and thickened near the base. Confocal microscopy indicates that their proximal and distal parts differ in light reflection and autofluorescence. Approximately 50 genera have atypically shaped setae: bifurcated, angled or swollen. These modifications are known in the basal part of prosomal setae *u′*, *ft′*, *ft″*, *d*, *v*, *bv*, *ve*, *sc* and caudal setae *h2*. We assessed the distribution of atypically shaped setae in Eriophyoidea and showed that they are scattered in different phylogenetic lineages. We hypothesized that the ancestral setae of eriophyoid mites were bifurcated before later simplifying into filiform setae. We also proposed that hypo-furcating setae are a synapomorphy that unites Eriophyoidea with Nematalycidae. We analyzed four new mitochondrial genomes of *Leipothrix,* the largest genus with bifurcated *d*, and showed that it is monophyletic and has a unique mitochondrial gene order with translocated trn*K*. We exclude *Cereusacarus juniperensis* **n. comb.** Xue and Yin, 2020 from *Leipothrix* and transfer five *Epitrimerus* spp. to *Leipothrix*: *L*. *aegopodii* (Liro 1941) **n. comb.**, *L. femoralis* (Liro 1941) **n. comb.**, *L. geranii* (Liro 1941) **n. comb.**, *L. ranunculi* (Liro 1941) **n. comb.**, and *L. triquetra* (Meyer 1990) **n. comb**.

## 1. Introduction

The superfamily Eriophyoidea (gall mites or four-legged mites) is a lineage of highly host-specific, permanent parasites of higher vascular plants. They have an unusual morphology for acariform mites, based on an elongate, vermiform body and only two pairs of legs. Eriophyoidea have moved to a completely different position within the system of Acariformes in recent years. For a long time, they were considered members of the cohort Eupodina, within the order Trombidiformes [1,2,3]. However, morphological and molecular phylogenetic studies performed in the last five years indicate that eriophyoids do not belong to Trombidiformes, placing them instead in a notably more basal position with soil mites of the family Nematalycidae, near the root of the Acariformes tree [4]. This long-term misinterpretation of Eriophyoidea was largely because of a number of homoplasies that were attributed too much importance, including plant feeding and a suite of interdependent paedomorphisms [2,4,5].

Reduced morphology, simplification, microscopic size and deficiency of phylogenetically informative characters are inherent characteristics of gall mites, affecting their systematics [1,2,6,7,8]. A large set of characters has been developed for classifying the supraspecific taxa of Eriophyoidea during the 20th century [9,10,11,12]. It includes such groups of traits as the following: chaetome (the set of all setae), position and shapes of setal tubercles, segmentation of legs, structure of the gnathosoma and female internal genitalia, and various characteristics of the opisthosomal cuticle, including the shapes of the opisthosomal annuli that may form various plates, protrusions, ridges and furrows. Based on these groups of characters, a higher classification and generic key of Eriophyoidea was developed by Amrine et al. [7] twenty years ago. Although this classification system is well designed for practical specialists and has been widely accepted by acarologists, it needs updating because many new supraspecific taxa of Eriophyoidea have been established, and progress in finding new morphological characters and understanding eriophyoid anatomy has been made since then [13,14,15,16]. Additionally, molecular phylogenetic studies of the last decade indicate that the current system of Eriophyoidea does not reflect the phylogeny and that many supraspecific taxa defined by morphology are artificial [8,14,17,18,19].

**Figure 1:**
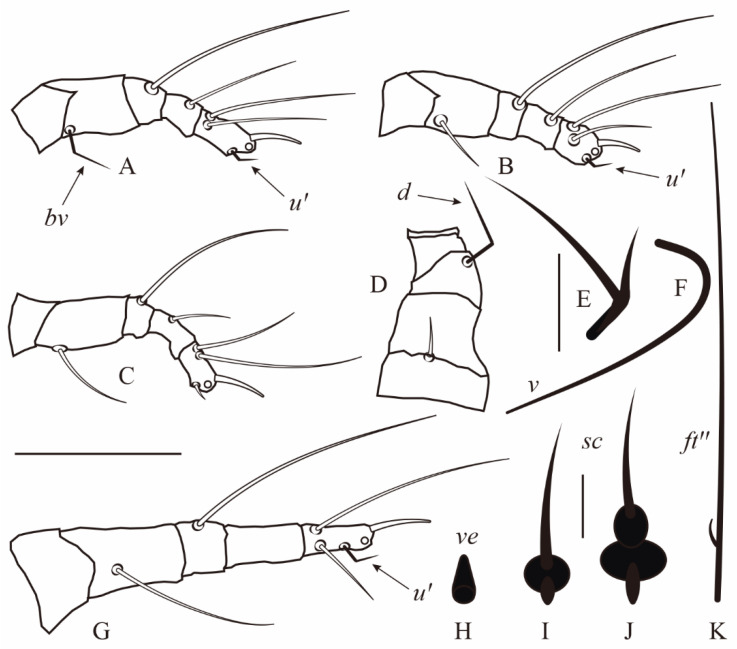
Atypically shaped setae in *Eriophyoidea* (redrawn from original descriptions). (**A**)—angled femoral seta *bv* I and tarsal seta *u′* I in *Notostrix trifida* Navia and Flechtmann, (**B**)—angled tarsal seta *u′* I in *N. miniseta* Navia and Flechtmann, (**C**)—*N. acuminata* Navia and Flechtmann (all leg setae commonly shaped), (**D**)—angled dorsal pedipalp genual seta *d* in *Propilus alternatus* Navia and Flechtmann, (**E**)—bifurcated pedipalp seta *d* in *Moraesia tau* Flechtmann, (**F**)—angled subapical pedipalp tarsal seta *v* in *Afrodialox dimorphopalpalis* Chetverikov and Craemer, (**G**)—angled tarsal seta *u′* II in *Propilus pellitus* Navia and Flechtmann, (**H**)—drop-shaped external vertical seta *ve* in *P. bactris* Reis and Navia, (**I**)—scapular seta *sc* with bulbose basal swelling in *Retracrus heliconiae* Ferreira and Navia, (**J**)—seta *sc* with two basal bulbose swellings in *R. pupunha* Reis and Navia, (**K**)—antaxial fastigial tarsal seta *ft″* in *Diptilomiopus floridanus* Craemer and Amrine. Scale bar: (**A**–**E**)—20 µm; (**F**–**H**)—5 µm. Note: in (**A**–**C**,**G**), empodium is not shown (only its basis is schematically depicted as a circle) for better observing setae *u′*. Scale bar: (**A**–**D**,**G**) = 20 µm; (**E**,**F**,**K**) = 5 µm; (**H**–**J**) = 5 µm.

**Figure 2:**
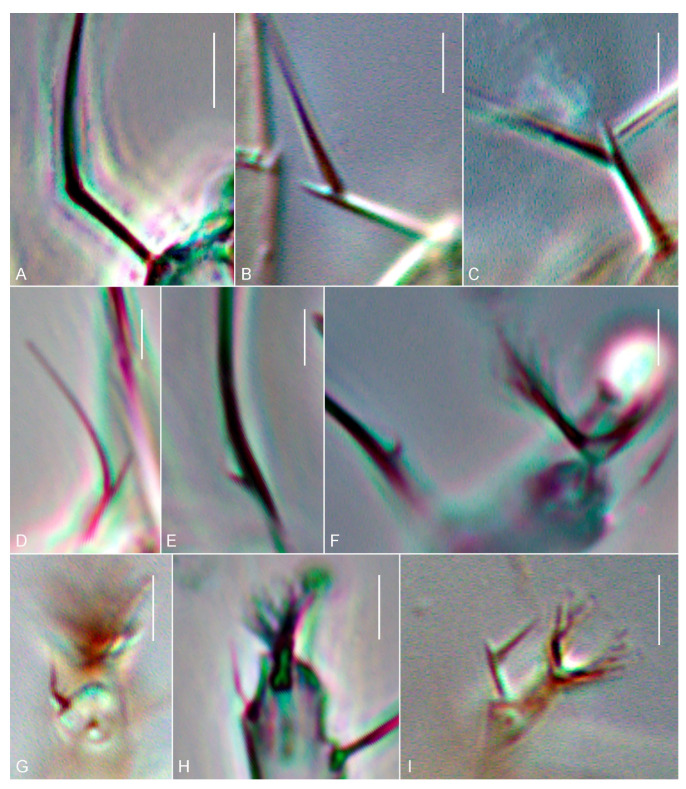
Light microscopy microphotographs of angled (**A**,**G**,**H**) or bifurcated (**B**–**F**,**I**) gnathosomal seta *d* (**A**–**D**) and tarsal setae *ft′* (**E**), *ft″* (**F**) and *u′* (**G**–**I**) in *Leipothrix triquetra* (Meyer) (**A**,**H**), *L. aegopodii* (Liro) (**B**), *L. ranunculi* (Liro) (**C**), *Tumescoptella aculeata* Chetverikov et al. (**D**), *Diptilomiopus floridanus* Craemer and Amrine (**E**,**F**), *Leipothrix knautiae* (Liro) (**G**), and *Tumescoptes dicrus* Meyer (**I**). Scale bar 2 μm.

The shape of setae is one of the characters used in the current systematics of gall mites. The shape of a certain seta is included as an obligatory trait in the diagnoses of some genera, but it does not discriminate any current suprageneric taxa [7]. As a rule, setae are smooth, filiform and unbranched in Eriophyoidea, but in some taxa, some setae may be atypically shaped, e.g., bifurcated or angled. According to our estimations, in 24 genera, gnathosomal seta *d* is bifurcated. In slide-mounted specimens, the smaller branch (the one-directed laterad, Figure 1E) is very often broken. Because of this, many species that have bifurcated seta *d* were described as having it “angled” (Figure 2A), e.g., various species of *Epitrimerus* were transferred to *Leipothrix* after this artifact was discovered [20,21]. When one branch of a bifurcated seta is rudimentary or completely suppressed, the seta also looks angled, e.g., pedipalp seta *d* in *Leipothrix solidaginis* Keifer [7] and the angled tarsal seta *u′* in various Phyllocoptinae and Sierraphytoptinae species [22,23,24,25,26,27].

*Leipothrix* Keifer is the largest phyllocoptine genus, characterized by bifurcated pedipalp setae *d*, three longitudinal opisthosomal ridges, and the absence of femoral setae *bv* I and II [7]. It comprises about 60 species, including a few with a taxonomic status that need confirmation because of unverified data on their chaetome [21]. Sexual dimorphism, generally poorly pronounced in Eriophyoidea [2,28], is prominent in *Leipothrix*, since their males are usually twice as small and move notably faster than females (P. Chetverikov and J. Amrine unpublished observations). All *Leipothrix* spp. are vagrant, typically living on the lower leaf surface. A few of them have been reported as causing rust, discoloration, slight deformation, wrinkling of leaves and witches’ broom, e.g., *L. dipsacivagus* (Petanović and Rector 2007) [29], but never causing true galls with a gall chamber (like a finger or pouch gall), erinea or bud galls that are characteristic of gall-forming Eriophyoidea [30,31]. Most species (~98%) are known from the Palearctic and predominantly inhabit herbaceous plants. Only three species were described in the Southern Hemisphere: *L. triquetra* (Meyer 1990) and *L. minidonta* (Meyer 1990) from ferns in South Africa, and *L. eichhorniae* (Keifer 1979) from the invasive water plant *Pontederia crassipes* Mart. in Brazil.

*Leipothrix* has a wide range of phylogenetically remote hosts. About a quarter of *Leipothrix* spp. (15 spp., ~28%) are associated with early-derivative plant clades (ferns—4 spp., magnoliids—3, and monocots—8). The remaining species (39 spp., ~72%) occur on eudicots and most of them (29 spp.) inhabit asterids. A single species, *Leipothrix juniperensis* Xue and Yin 2020, is known from conifers. According to the original description [32], this species does not fit the diagnosis of *Leipothrix* because it has femoral setae *bv* I and II (absent in *Leipothrix*).

GenBank data on *Leipothrix* include 215 sequences (accessed on 1 August 2023). Among them, 213 sequences belong to *Leipothrix* sp. and *L. juniperensis* from China. They include fragments of *COX1*, *18S* and *28S* genes and one complete mitochondrial genome (KX027362) [18]. Two sequences belong to *L. liroi* (ITS1-5.8S, MH522408) from *Primula* sp. from Iran and *Leipothrix* sp. (D1D2 28S, KT070277) from the fern *Cheilanthes viridis* from South Africa [17].

In this paper, we aim to (1) assess the distribution of bifurcated and other atypically-shaped setae across eriophyoid genera; (2) test the monophyly of the genus *Leipothrix* and the whole group of eriophyoid genera possessing such setae; (3) clarify the phylogenetic position of *L. juniperensis* via molecular markers; and (4) investigate the organization of the mitochondrial genome in *Leipothrix* in order to reveal if *Leipothrix* spp. shares a common mitochondrial gene order (MGO) and if this MGO deviates from those in other eriophyoid taxa.

## 2. Materials and Methods

In order to review the distribution of atypically shaped setae in Eriophyoidea, we performed an extensive literature search and screened original descriptions of eriophyoid taxa from our libraries and various well-illustrated regional and world catalogs containing morphological drawings of eriophyoids [7,23,24,25,26,27,33,34]. We also examined eriophyoid mites under light microscopy (LM) (differential interference contrast (DIC) and phase contrast (PC)), using a Leica DM2500 microscope, the slide-mounted eriophyoids from the Acarological Collection of ZIN RAS, focused mainly on the genera with atypically shaped setae. In order to investigate the behavior of eriophyoid setae under illumination of a blue laser (405 nm), we analyzed confocal laser scanning microscopy (CLSM) stacks of various eriophyoid taxa, obtained using a spectral confocal and multiphoton system Leica TCS SP2 with objectives 63× N.A. 1.4–0.60 Oil lBL HCX PL APO and 40× N.A. 1.25–0.75 Oil CS HCX PL APO, at an excitation wavelength of 405 nm, and an emission wavelength range of 415–750 nm, at 10–20% intensity from our previous studies [35,36,37,38].

For molecular studies, we obtained sequences of two genes (*COX1* and *D1D2 28S*) of five phyllocoptines that have bifurcated setae *d* and complete mitochondrial genomes of four *Leipothrix* spp. (Table 1). For this purpose, we used the same methodology and protocols for DNA extraction, library preparation, PCR, genome sequencing, assembly and annotation as described in [39,40]. Three sequence datasets (*Cox1*, *28S* and mitogenomic) were made for molecular phylogenetic analyses. The *Cox1* dataset included 1409 unique sequences of gall mites from GenBank. They were translated into amino acids and aligned in MAFFT [41,42] with default adjustments, resulting in the final alignment consisting of 423 amino acid positions. For creating the *28S* dataset, we blasted sequence OR416172 of *L. aegopodii* against Eriophyidae and filtered the sequences of 45% coverage. The remaining 166 sequences were aligned and modified as described in [40]. The mitogenomic dataset included 11 complete sequences of mitochondrial genomes, among them 7 sequences from Genbank [18,32,40,43,44,45] and 4 sequences obtained in this study (Table 1).

Maximum likelihood analyses were conducted in IQ-TREE 2 [46]. For gene evolution, the GTR + F + I + G4 model was selected for the *28S* datasets and the mtART + R3 model was selected for the *Cox1* dataset using ModelFinder [47], as implemented in IQ-TREE 2 based on the Bayesian Information Criterion. Branch support values were generated from the ultrafast bootstrap approximation (UFBoot), with 10,000 bootstrap alignments, 10,000 maximum iterations and a minimum correlation coefficient of 0.99. Values of a single branch test (SH-like approximate likelihood ratio test, SH-aLRT) with 10,000 replicates and ultrafast bootstrap support (UFBS) were labeled on the maximum likelihood (ML) trees.

For the mitogenomic analysis, mitochondrial rRNA (*12S* and *16S*) and protein genes (translated into amino acids) were aligned using MAFFT, using an E-INS-I algorithm for 12S and 16S genes and a G-INS-i algorithm for the protein genes. The resulting alignments were modified using Gblocks [48,49], as described in [40]. Sequences of the ATP8 gene were excluded because Gblocks failed to find reliable blocks in the alignment of this gene. For gene evolution, the GTR + F + I + G4 model was selected for the merged *12S* + *16S* datasets, mtZOA + F + G4 was selected for the merged *ATP6* + *COX2* + *COX3* + *NAD1* + *NAD2* + *NAD3* + *NAD4* + *NAD4L* + *NAD5* + *NAD6* datasets, and the mtART + I + G4 model was selected for the merged *COX1* + *CYTB* dataset using ModelFinder. All other steps of the analysis were similar to those described for the *Cox1* and *28S* analyses. Sequences of two phytoptid taxa (*Fragariocoptes* and *Retracrus*) from GenBank were used for rooting the trees.

## 3. Results

### 3.1. Distribution of Atypically Shaped Setae in Taxa of Eriophyoidea

Except empodia and solenidia (not discussed in this paper), the setae in eriophyoid mites are usually unbranched and filiform [1,2,7]. In ~50 genera from three families (Phytoptidae s.str., Eriophyidae s.str. and Diptilomiopidae), some setae are of an atypical shape: angled, bifurcated or with swellings (Figure 1, Table 2). These modifications are always in the basal part of the setae. Atypical setae are present mostly on the prosoma and include leg setae *u′*, *bv*, *ft′* and *ft″,* gnathosomal setae *d* and *v*, and prodorsal shield setae *ve* and *sc*. The listed leg and gnathosomal setae may be angled or bifurcated (bearing an extra branch), whereas when prodorsal shield setae are modified, they have one or two bulb-like swellings near the base (Figure 1). A single phyllocoptine species (*Leipothrix nagyi* Ripka et al. 2020) has modified *h2* [50], the paired setae that are located in the caudal part of opisthosoma in all Eriophyoidea [1].

Setal bifurcations and angulations are the most common in Eriophyoidea. They have been reported in many genera of the two subfamilies of Eriophyidae (Phyllocoptinae and Nothopodinae), in a few genera of Diptilomiopidae and in two genera of Phytoptidae s.str. (Table 2). Gnathosomal seta *d* and leg seta *u′, ft′* and *ft″* may be modified in both of these two ways. Angled *bv* is known only in *Notostrix trifida* Navia and Flechtmann 2003 and angled *ft′* and *ft″* in *Neodicrothrix grandcaputus* Yuan and Xue 2019. Branched *ft′* and *ft″* is only known in two species of *Diptilomiopus* (*D. floridanus* Craemer and Amrine 2017 (Figure 1A,K) and *D. careyus* Qin et al. 2019) and branched *h2* in one species of *Leipothrix* (*L. nagyi* Ripka et al. 2020, fig. 1 PM in [50]). Angled gnathosomal *v* has been reported in a few genera of Diptilomiopidae (Figure 1F, Table 2). Drop-shaped setae and those with basal bulb-like swellings are known in sierraphytoptine genera *Propilus* (Figure 1H) and *Retracrus* (Figure 1I,J). No species with atypical setae have been registered in the conifer-associated lineage Nalepellidae except *Nalepella* sp., which has bulbous swellings near the bases of setae *sc* when observed under SEM (R. Petanović, personal communication, July 2011).

Among eriophyoid genera, there are a few in which atypical setae are present in some species and absent in others. For instance, in *Notostrix*, most species have no atypically shaped setae, a few species have angled *u′* and in one species (*N. trifida*), *bv* and *u′* are angled (Figure 1A–C). *Leipothrix* is the largest eriophyoid genus (~60 species), all members of which possess bifurcated *d*. Remarkably, at least seven *Leipothrix* spp. from our collections (*L. aegopodiae*, *L. convallariae*, *L. femoralis*, *L. geranii*, *L. jaceae*, *L. knautiae*, *L. ranunculi*, *Leipothrix* sp. and *L. triquetra*) have angled *u′*, missed by previous authors (Figure 2G,H). Finally, most small and monotypic eriophyoid genera with atypical setae inhabit palms (Arecaceae) in South America and Africa and different subtropical dicotyledonous trees in Asia.

### 3.2. Microscopic Observations

Under a stereomicroscope (magnification about ×20–×80), the setae of eriophyoid mites are tiny hair-like structures, usually of distinct black color. This color is especially well seen in some Phytoptidae s.l. (e.g., in *Nalepella* and *Novophytoptus* with stout *sc*, in *Oziella* with long *c1*) and in various species of Eriophyidae s.l. that have long opisthosomal setae *c2*, *d*, *h2*. Some species of the listed phytoptid genera are capable of moving their long setae *sc* or *c1* with an amplitude of up to ~50 degrees, apparently due to strong opisthosomal musculature operating the setal bases (I.G. Bagnjuk personal communication, 1996; P.E. Chetverikov, unpublished observations, 2004, 2020).

**Figure 3:**
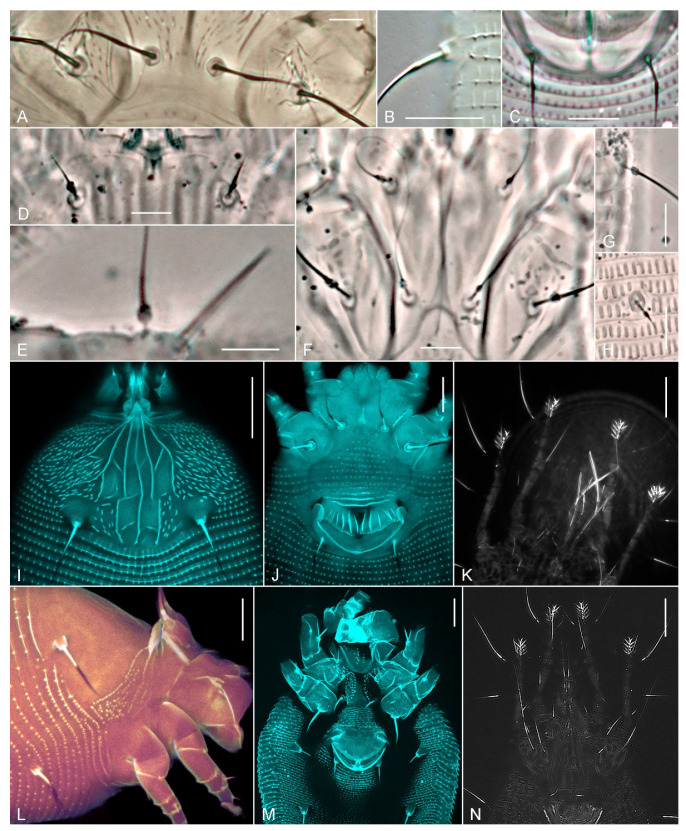
Conventional PC LM (**A**,**C**–**H**) and DIC LM (**B**) images showing the transition area between the basal and distal parts of different setae in selected eriophyoids. (**A**)—coxal setae *1a* and *2a* of *Leipothrix jaceae* (Liro), (**B**,**C**)—setae *f* (**B**) and *3a* (**C**) in *L. knautiae* (Liro), (**D**–**H**)—subspherical clusters stuck to the area between the proximal and distal parts of setae *sc* (**D**), *ft″* (**E**), *1a*, *1b*, *2a* (**F**), *f* (**G**) and *e* (**H**) in *Oziella liroi* (Roivainen). CLSM images (**I**–**K**,**M**,**N**)—maximum intensity projections, (**L**)—volume rendering) show differences in autofluorescence (**I**,**J**,**L**,**M**) and light reflection (**K**,**N**) between the distal and proximal parts of eriophyoid setae when illuminated with blue laser (405 nm). (**I**)—*Aceria acroptiloni* Shevchenko and Kovalev, (**J**)—*Metaculus rapistri* Carmona, (**K**)—*Phytoptus chamaebatiae* Keifer, (**L**)—*Phyllocoptes bilobospinosus* Chetverikov, (**M**)—*Nalepella tsugifoliae* Keifer, (**N**)—*Setoptus pini* Boczek. Scale bar: (**A**–**H**) = 5 μm, (**I**–**N**) = 10 μm.

Under conventional light microscopy (magnification ×1000), all common filiform setae in most slide-mounted specimens from our collections that we observed consist of two parts, a short proximal part and long distal part, often forming an obtuse angle that is very close to 180° (Figure 3B,C). Because of this slight curvature, under CLSM adjusted to capture the reflected light of a laser, the reflection of the proximal and distal parts of a seta is different. Only one of these parts (usually the distal one, depending on the position of the specimen on the slide) reflects the light at the right angle to be captured by CLSM, whereas the other part does not produce reflection and is unobservable using the “reflection mode” of CLSM (Figure 3K,N).

In our collections, we have specimens of Pentasetacidae, Phytoptidae s.l. and Eriophyidae s.l., slide mounted under suboptimal conditions, allowing the dust of the air or other extraneous material to occur in the mounting medium. In these cases, the extraneous objects tend to form clusters in the form of dark drops attached to the curved area between the basal and distal parts of setae (Figure 3D–H). Under CLSM applied for capturing the emission light when illuminating mites with a blue laser (405 nm), the proximal part of all setae exhibits very strong autofluorescence, whereas the distal part produces no signal (Figure 3I,J,L,M).

Under conventional DIC LM and PC LM, the proximal and distal parts of eriophyoid setae differ by birefringence and thickness. The proximal (most basal) part always looks like a hollow, linear structure with dark outlines and lighter medial content that is green or blue depending on the applied objective. In long setae, especially in coxal setae *2a*, genital setae *3a*, and opisthosomal setae *c2*, *d* and *f*, the proximal part is often followed by a more or less distinct but always very tiny thickening (Figure 3A–C). It marks the curvature zone mentioned above and continues into a hair-like distal part that is dark and solid. Under SEM and LT-SEM, these tiny elements of setal morphology are rarely seen. They may be hidden under the layer of the sputter coating or they snap off because of the pressure changes. This may explain why bifurcating setae often end up looking angled under SEM.

### 3.3. Molecular Phylogenetics: Blast Searches and COX1 and 28S Analyses

Blast searches for *D1D2 28S* sequences of the four new sequences of *Leipothrix* (Table 1) against Eriophyidae returned, as the best hit, the sequence KT070277 of *Leipothrix* cf *triquetra* from *Cheilanthes viridis* from South Africa, with 99–100% coverage and 90–91% identity. Sequences of *Leipothrix juniperensis* were absent in the list of the 100 most similar *28S* sequences returned by Blast for the four *Leipothrix* spp.

Blast searches for *COX1* (MZ274920) and *28S* (MZ289016) sequences of *Leipothrix juniperensis* except conspecific sequences returned various sequences of phyllocoptines associated with gymnosperms (e.g., *Epitrimerus sabinae*, *Phyllocoptruta platycladusa*, *Stenacis thunbergii)*, all of them without bifurcated setae. Inclusion of the new *COX1* and *28S* sequences of the four *Leipothrix* spp. in the Blast searches did not change the result for *L. juniperensis*.

**Figure 4:**
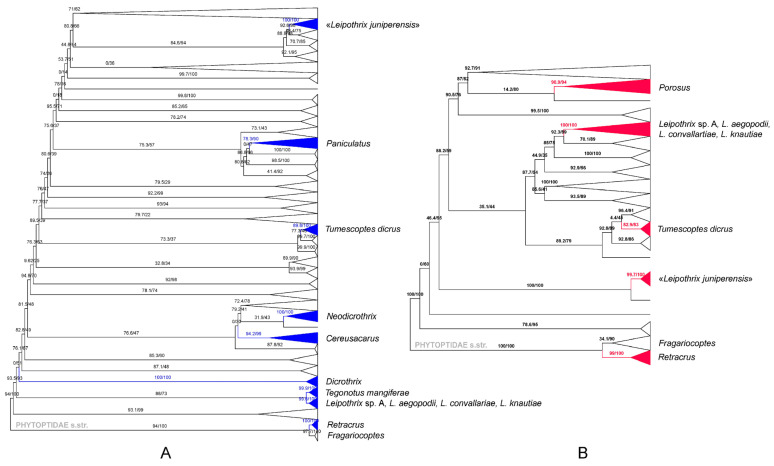
Two maximum likelihood trees showing the relative position of eriophyoids of the genera *Dicrothrix*, *Leipothrix*, *Neodicrothrix*, *Porosus*, *Paniculatus*, *Tegonotus*, *Tumescoptes* and *Retracrus,* having a bifurcated pedipalp seta *d*. Clades containing these species are collapsed and colored blue in the *COX1* tree (1409 sequences, 423 amino acids, (**A**)) and red in the *28S* tree (166 sequences, 1648 nucleotide positions, (**B**)).

Maximum likelihood analyses of *COX1* (Figure 4A) and *28S* (Figure 4B) sequences produced poorly resolved trees with many small, well-supported clades, which is typical when using these genes for analyzing large sets of sequences of Eriophyoidea. The eriophyoid taxa with bifurcated pedipalp seta *d* included in our *COX1* (*Cereusacarus*, *Dicrothrix*, *Leipothrix*, *Neodicrothrix, Paniculatus, Retracrus, Tegonotus mangiferae, Tumescoptes*) and *28S* (*Leipothrix*, *Porosus*, *Retracrus*, *Tumescoptes*) datasets are scattered across the trees, such that they are among distantly related clades. In all analyses, the four new sequences of *Leipothrix* (Table 1) form a highly supported clade, indicating the monophyly of this group of species (“true *Leipothrix*”, t*L*). *COX1* analysis revealed a moderately supported clade: t*L* + *T. mangiferae*. Sequences of *Leipothrix juniperensis* (“false *Leipothrix*”, f*L*) were not grouped with t*L* or any other taxa with a bifurcated pedipalp *d* (including *Cereusacarus*) and cluster with sequences of various conifer-associated phyllocoptines (*Epitrimerus sabinae*, *Phyllocoptes taishanensis*, *Phyllocoptruta platycladusa*, *Glossilus* sp.).

### 3.4. Mitogenomics

**Figure 5:**
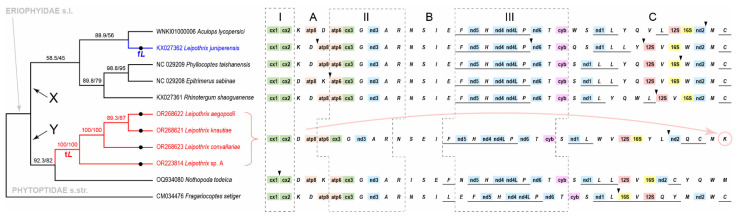
Maximum likelihood phylogeny (12 mitochondrial protein genes, *12S*, and *16S*) of Eriophyoidea (left) and gene orders in the mitochondrial genomes included in the analysis (right). New sequences of *Leipothrix* spp. (“true *Leipothrix*”, t*L*) are colorized red and *L. juniperensis* (“false *Leipothrix*”, f*L*) is colorized blue. Branch labels are the following: SH-aLRT support (%)/ultrafast bootstrap support (UFBS, %). Black circles (•) indicate taxa with bifurcated pedipalp seta *d*. The constant blocks (**I**–**III**) and variable zone (**A**–**C**) of mitochondrial genes in eleven eriophyoid mite species are indicated. Translocation of the trn*K* gene from zone A to zone C is shown by the pink arc-shaped arrow. Black arrowheads point to the position of control regions. Genes located on the negative chain of mitochondrial DNA are underlined. Notations: cx—Cytochrome c oxidase (green), atp—ATP synthase (orange), nd—NADH dehydrogenase (blue), cyb—Cytochrome b (purple), 12S and 16S—rRNA genes (red and yellow), X and Y—two clades of Eriophyidae s.l. recovered in this analysis.

Four new sequences of the complete mitochondrial genomes of *Leipothrix aegopodii*, *L. convallariae*, *L. knautiae*, and *Leipothrix* sp. A were assembled and annotated (Figure 5, Table 3). The average size of a mitogenome is 13,593 ± 110 bp. Each mitogenome includes 37 similarly ordered genes (13 protein-coding genes, 2 rRNA genes, 22 tRNA genes and 1 control region), 10 of which are located on the negative chain. Protein-coding genes terminate with stop codons TAA (67.31%) or TAG (26.92%), except genes *NAD3* (in *L. knautiae* and *L. convallariae*) and *NAD5* (in *L. convallariae*), which terminate with mononucleotide T. The control region (*CR*) in all mitogenomes is flanked by genes trnL and *NAD2* and varies in size from 38 bp in *L. convallariae* to 184 bp in *L. knautiae* (Table 3). In *Leipothrix*, sp. A and *L. knautiae* the *CR* has complementary poly-G and poly-C fragments, forming a large D-loop of ~100 bp. The four new mitogenomes of *Leipothrix* spp. comprise the same constant blocks I, II, III and variable zones A,B,C (Figure 5), recently defined in other published mitogenomes of Eriophyidae [18,32,40,43,44,45]. They share the following unique traits: (1) the trn*K* gene precedes the *COX1* gene, (2) two tRNA genes coding leucine are located on different chains of mitochondrial DNA, (3) a cluster of tRNA genes W–V precedes the *12s* rRNA gene, (4) genes *16s* rRNA and *COX1* flank a group of uniquely arranged genes and the control region Y–L–(*CR*)–*NAD2*–Q–C–M–K (genes located on the negative chain are underlined).

The only mitogenome in GenBank assigned to the genus *Leipothrix* (KX027362.1, *L. juniperensis*) [18] does not have the traits listed above (Figure 5). It has trn*K* flanked by *COX2* and trn*D*, both trn*L* genes located on the negative chain, genes trn*V* and trn*W* flanking the *16s* rRNA gene, and a gene cluster *W*–*NAD2*–*M*–*C* lacking a *CR* and situated between its *16s* rRNA and *COX1* genes. The MGO in this species is closest to that in *Phyllocoptes taishanensis* (NC_029209) [43], except the position of the trn*Q* gene and the number and position of the *CR*: a single *CR* between trn*Y* and *12S* rRNA in *P*. *taishanensis* vs. three *CR* located in zones A, III and C in *L. juniperensis* (Figure 5).

Maximum likelihood analysis of the mitogenomic dataset produced a poorly resolved tree, comprising two clades—X and Y (Figure 5). The “true *Leipothrix*” is monophyletic. *Leipothrix juniperensis* and t*L* are nested within clades X and Y (correspondingly).

## 4. Discussion

**Is setal bifurcation an ancestral character state in Eriophyoidea?** Our observations indicate the following: (a) common filiform setae in Eriophyoidea are usually slightly bent near the base and may be thickened, which is only noticeable with high-level optics; (b) dust particles and other extraneous material occurring in the mounting medium tend to form clusters around this swelling; and (c) proximal and distal parts of setae differ in light reflection and autofluorescence. We also showed that in Eriophyoidea, all modifications always happen in the basal part of a seta and they are scattered throughout different phylogenetic lineages of Eriophyoidea. These data suggest that eriophyoid setae are more complex than previously thought. We hypothesize that ancestrally, all setae in eriophyoid mites were bifurcated and later simplified into filiform setae, with one of the two setal branches shortened or completely reduced, the latter resulting in an angled seta. This “simplification” scenario agrees with the general reduction trend in Eriophyoidea. It is also more parsimonious than the alternative scenario, which would require that in many phylogenetically unrelated lineages, the simple filiform setae have transformed into bifurcated or angled setae in parallel. Non-monophyly of the groups of eriophyoid taxa possessing different, atypically shaped setae has been revealed by *COX1* and *28S* molecular phylogenetics (Figure 4) and other analyses that include larger datasets [8,18,19]. This agrees with the hypothesis on the plesiomorphic nature of bifurcated setae in Eriophyoidea.

**Synapomorphic status of bifurcating setae.** Based on a consensus of molecular and morphological phylogenetic analyses, there is now very strong support for a close relationship between Eriophyoidea and Nematalycidae [4,5,44,51,52,53]. The bifurcating form of setae in Eriophyoidea provides additional evidence for this affiliation. Bifurcating and trifurcating setae are rare and unusual structures in Acariformes. The vast majority of mite setae are either unbranched or have more than three branches (hyper-furcating). But in Nematalycidae, almost all setae are unbranched, bifurcating or trifurcating [54,55,56,57,58]. If bifurcated setae are the ancestral condition in Eriophyoidea, hypo-furcating setae (herein defined as setae with two or three branches) represent another potentially important synapomorphy that unites Eriophyoidea with some or all Nematalycidae.

**Figure 6:**
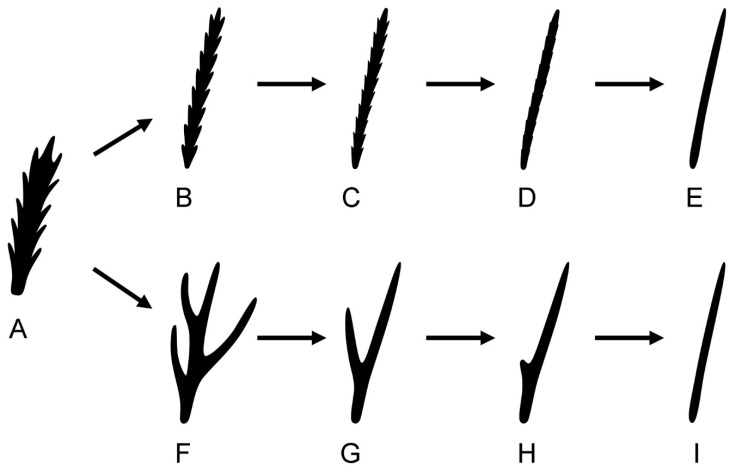
Two different evolutionary pathways from a seri-furcating seta to an unbranched seta. (**A**)—typical seri-furcating seta of a basal endeostigmatid mite, representing a hypothetical plesiomorphic form of seta for Acariformes; (**B**–**E**)—trombidiform pathway; (**F**–**I**)—nematalycid-eriophyoid pathway.

**Typology and evolution of furcating setae.** The hypo-furcating form of setae in Nematalycidae and Eriophyoidea appears to represent a derived form that is entirely distinct from the hyper-furcating form that occurs in Trombidiformes and most lineages within Endeostigmata (a basal grade that is probably paraphyletic to Trombidiformes, Oribatida and Eriophyoidea [34,52,53]). By far the most common form of hyper-furcating seta is a serial branching (seri-furcating) seta, in which the branches form rows along a single, central stem (Figure 6A–D). Fractal or dendritic (tree-like) branching (dendro-furcating) represents another form of branching seta, in which branches subdivide into further branches [59]. However, that type of seta is very rare in mites. In some Endeostigmata, the stems of seri-furcating setae on the hysterosoma often swell distally to form bulbous, club- or wedge-shaped structures [5,59,60], whereas in Trombidiformes, the stems of seri-furcating setae are usually filiform [3].

Due to its dominance among basal families of Endeostigmata [61], the seri-furcating seta likely represents the plesiomorphic form of seta for Acariformes (Figure 6A). In addition to the unbranched form of seta, seri-furcating setae are also very common in Trombidiformes [62,63,64,65]. Whereas in some species of Trombidiformes the setules (branches) of seri-furcating setae are long, e.g., in *Allothrombium fuliginosum* (Hermann) [66] (Figure 338E), in others, the setules are so short so as to be vestigial and almost indiscernible, e.g., in *Abrolophus rubipes* Trouessart [66] (Figure 338F). Hypo-furcating setae are extremely rare in Trombidiformes. The relatively common unbranched form of seta in this lineage (e.g., *Metatarsonemus* [67]) is readily explained by the parallel reduction and eventual loss of the many setules of seri-furcating setae (Figure 6A–E), and so setae with extremely short setules (Figure 6D) appear to represent a transitional form between setae with long setules (Figure 6A) and unbranched setae (Figure 6E). Notably, some trombidiform species simultaneously bear smooth and unbranched setae in addition to seri-furcating setae with extremely short setules [67].

Some endeostigmatids, such as Micropsammidae, have seri-furcating setae with a low number of setules (Figure 6F), whereas the trifurcating, bifurcating and unbranched setae of Nematalycidae and Eriophyoidea have too few branches to be seri-furcating. Due to this absence of seri-furcating setae, the unbranched setae of eriophyoids and nematalycids cannot be readily explained by the parallel reduction in many setules. Instead, individual setules have probably been reduced and lost sequentially (Figure 6F–I), such that the number of setules gradually diminishes until it reaches zero, resulting in an unbranched seta (Figure 6I). In some or perhaps all cases, individual setules may have gradually diminished in length, so that only a single vestigial stump or projection remains (Figure 6H) before any trace of a setule is completely gone (Figure 6I). In Nematalycidae and Eriophyoidea, bifurcating setae are often observed that have this stump-like vestige of a setule (Figure 6H). These setae are referred to as semi-bifurcating in the description of the nematalycid, *Osperalycus tenerphagus* Bolton and Klompen [57]. The exact same type of setae is especially abundant on the leg segments of the nematalycid, *Psammolycus delamarei* Schubart [58].

Therefore, the unbranched form of seta in eriophyoids and nematalycids appears to have arisen in a completely different way from the unbranched form of seta in trombidiform mites. Moreover, the absence of seri-furcation and the presence of hypo-furcation in both Eriophyoidea and Nematalycidae further weakens the case for the placement of Eriophyoidea within Trombidiformes.

**Taxonomic status of *Leipothrix juniperensis* and monophyly of *Leipothrix*.** Recently, Yin et al. [19] examined the accuracy of molecular delimitation methods (BIN, ABGD, ASAP, GMYC and mPTP) and advocated for employing multiple analytical approaches to aid correct species delimitation in gall mites. A priori, the effectiveness of these methods depends on the availability of the carefully curated sequences that are stored in public databases (e.g., GenBank), which have unique numbers and are assigned to a peer-reviewed paper verifying the origin of the sequences [68]. With new submissions, the number of erroneous sequences of Eriophyoidea uploaded to GenBank has been increasing every year [40], which makes it difficult to obtain correct molecular cladograms using data from this database. For instance, in this study, we found that the sequence MW251739 of a gall mite *Acalitus vaccinii* belongs to a crustacean (blastx 99.4% similarity with QIZ03131 of *Campylaspis sulcata* [69]), and eriophyoid sequences MZ483068 of *Leipothrix* sp. 1 XFX-2017 [18] and KM111096 of *Cheiracus sulcatus* [70] are 100% identical, meaning a wrong generic assignment.

Four GenBank sequences (MZ255376, MZ274920, MZ289016, MZ326598) are assigned to *Leipothrix juniperensis* Xue and Yin, 2020 in [32]. It was described from the samples containing specimens of *Epitrimerus sabinae* s.l. Xue and Hong 2005 and collected from *Juniperus chinensis* L. (Cupressaceae) from various locations in China. The morphological concept of *L. juniperensis* was tested using molecular methods, including DNA-based species delimitation, phylogenetics, haplotype network and comparative mitogenomics [32]. Yet, according to the original description, it does not fit the morphological diagnosis of *Leipothrix*.

Among eriophyoid genera, “*juniperensis*” is morphologically closest to *Cereusacarus* Xue et al., also described from China [71]. They share the bifurcated pedipalp seta *d*, legs and opisthosoma with usual series of setae (including *bv* I and II present, contrary to *Leipothrix*, in which *bv* are absent), as well as opisthosoma with the middorsal ridge ending before the lateral ridges. However, “*juniperensis*” differs from *Cereusacarus* in some body shape characteristics. In *Cereusacarus*, the middorsal opisthosomal ridge ends in a furrow, the first five dorsal annuli are almost as wide as the prodorsal shield, with the next annuli abruptly narrower, and the dorsal and ventral annuli are not differentiated [71]. In the protogyne of “*juniperensis*”, it is uncertain whether the middorsal opisthosomal ridge ends in a furrow. The annuli taper gradually from the prodorsal shield posteriad, and the opisthosomal annuli are differentiated into broader dorsal semi-annuli and narrower ventral semi-annuli [32]. Since, morphologically, “*juniperensis*” much more closely resembles *Cereusacarus* than *Leipothrix*, we exclude it from *Leipothrix* and provisionally transfer it to *Cereusacarus*: *C. juniperensis* (Xue and Yin, 2020 in [32]) **comb. nov.** It should be noted that sequences of *Cereusacarus* and “*juniperensis*” do not cluster together in our *COX1* tree (Figure 4). Therefore, the proper generic placement of “*juniperensis*” needs further testing.

Since Amrine et al. [7] revised the morphological concept of the genus *Leipothrix* and stated that the presence of a bifurcated pedipalp seta *d* and absence of femoral setae *bv* I and II are obligatory characteristics of this genus, the number of species assigned to this genus significantly increased due to the discovery of new species and the transfer of some older species from other genera into *Leipothrix* [21,50,72,73,74,75,76,77,78,79,80,81,82,83,84,85,86,87]. In this study, we sequenced four species of *Leipothrix* and verified the generic assignment of *L*. *aegopodii* (Liro 1941) **n. comb.**, *L. femoralis* (Liro 1941) **n. comb.**, *L. geranii* (Liro 1941) **n. comb.**, *L. ranunculi* (Liro 1941) **n. comb.** and *L. triquetra* Meyer 1990 **n. comb.** by light microscopy. Besides basic morphological uniformity, molecular phylogenetics indicates that species of this genus form a highly supported clade t*L* in *Cox1*, *28S* and mitogenomic trees (Figure 4 and Figure 5). Finally, the unique gene order discovered in four t*L* mitogenomes (Figure 5) confirms the monophyly of *Leipothrix* and provides an additional basis for the exclusion of “*juniperensis*” from this genus.

Overall, our study indicates the importance of carefully examining the chaetome of eriophyoid mites for correct generic assignments and calls for the curation of sequences after they have been uploaded to GenBank. It also points in several new directions that would contribute to a further understanding of the phenomenon of atypically shaped setae in Eriophyoidea and their evolution: (1) reexamination of old eriophyoid taxa in order to reveal the true morphology of their setae; (2) comparative studies of the chaetome of Eriophyoidea and Nematalycidae, including additional investigation of the fine structure of their setae with the aid of various microscopic techniques, including transmission electron microscopy; and (3) taxonomic revisions, molecular phylogenetics and mitogenomics of eriophyoid genera with atypically shaped setae, especially palm associated genera, e.g., *Notostrix*, *Propilus, Tumescoptes* and *Retracrus.*

## Figures and Tables

**Table 1:** Collecting data and GenBank accession numbers for five eriophyoid mite species.

Mites Species	Collecting Data	GB Accession Numbers		
		*Cox1*	*D1D2 28S*	Mitogenome
*Leipothrix aegopodii* (Liro 1941)	RUSSIA: Novgorod area, near vil. Peredolskaya, right bank of the river Luga, 7 July 2018, 58°29′11.4″ N, 30°20′14.3″ E, from lower leaf surface of *Aegopodium podagraria* L. (Apiaceae), coll. P.E. Chetverikov	OR414018	OR416172	OR268622
*Leipothrix knautiae* (Liro 1942)	RUSSIA: Leningrad area, Gatchina distr., vil. Vyritza, 59°23′50.0″ N, 30°17′41.6″ E, 21 September 2019, from lower leaf surface of *Knautia arevensis* (L.) Coult. (Dipsacaceae), coll. P.E. Chetverikov	OR414015	OR416171	OR268621
*Leipothrix convallariae* (Liro 1943)	LATVIA: Salasgriva Prov., pine forest between highway A1/E67 and Baltic sea, 26 July 2019, 57°38′16.3″ N 24°22′23.4″ E, from lower leaf surface of *Convallaria majalis* (L.) (Asparagaceae), coll. P.E. Chetverikov	OR414017	OR416173	OR268623
*Leipothrix* sp. A	USA: West Virginia, Monongalia Co, near Morgantown, 1 July 2017, 39°38′54.9″ N, 79°52′04.2″ W, from lower surface of fronds of *Athirium filix-femina* (L.) Roth (Athyriaceae), coll. J. Amrine and P.E. Chetverikov	OR414016	OR416170	OR223814
*Tumescoptes dicrus* Meyer 1992	SOUTH AFRICA: Cape Town, near Kirstenbosch National Botanical Garden, 33°59′09.4″ S 18°26′01.7″ E, 12 November 2016, inside folded young fronds of *Phoenix reclinata* (Arecaceae), coll. P.E. Chetverikov, C. Craemer, S. Neser	OR414014	OR416174	-

**Table 2:** Distribution of atypically shaped setae in genera of Eriophyoidea. Asterisks (*) indicate the genera with all members possessing a certain atypically shaped seta.

Seta	Shape	Eriophyoid Genera Containing Species with Atypically Shaped Setae
pedipalp *d*	angled (Figure 1D and Figure 2A)	Phytoptidae s.str.: Sierraphytoptinae: *Propilus alternatus*; Eriophyidae: Acritonotus *, Paniculatus *, *Reginesus* *, *Spinacus*, *Pseudotagmus* *
	bifurcated (Figure 1E and Figure 2B–E)	Phytoptidae s.str.: Sierraphytoptinae: (*Propilus, Retracrus*); Eriophyidae s.str.: Phyllocoptinae: *Acaphylla*, *Adenoptus*, *Asetidicrothrix* *, *Athrix* *, *Bangphracarus* *, *Calpentaconvexus* *, *Cereusacarus* *, *Chiacaphyllisa* *, *Dicrothrix* *, *Euteria* *, *Glabrisceles* *, *Kraducarus* *, *Kosacarus* *, *Leipothrix* *, *Moraesia* *, *Mangophyes* *, *Navia* *, *Neodicrothrix* *, *Porosus* *, *Protumescoptes* *, *Tegonotus*, *Tegophyes* *, *Vareeboona* *, *Tumescoptella* *
*v*	angled (Figure 1F)	Diptilomiopidae: Afrodialox *, *Apodiptacus*, *Asetacus*, Neodialox *, Dialox *, *Hyborhinus*, Catarhinus *, Vimola *
*u′*	angled (Figure 1A,B,G and Figure 2G,H)	Phytoptidae s.str.: Sierraphytoptinae: *Propilus*, *Retracrus* Eriophyidae s.str.: Phyllocoptinae: *Aculus*, *Adenoptus*, *Heterotergum*, *Leipothrix*, *Notostrix*, *Platyphytoptus*, *Reginesus* *, *Thacra* *, *Tumescoptella* *, *Pseudotagmus* * Eriophyidae s.str.: Nothopodinae: *Catachela*, *Cosella*, *Dechela*, *Juxtacolopodacus* *, *Neocosella*
	bifurcated (Figure 2I)	Eriophyidae s.str.: Phyllocoptinae: *Tumescoptes*, *Notostrix*, *Euterpia* *
*ft′*, *ft″*	with short additional branch (Figure 1K and Figure 2E,F)	Diptilomiopidae: Diptilomiopinae: *Diptilomiopus careyus* and *D. floridanus*
	angled	Eriophyidae s.str.: Phyllocoptinae: *Neodicrothrix grandcaputus*
*bv*	angled (Figure 1A)	Eriophyidae s.str.: *Notostrix trifida*
*h2*	bifurcated	Eriophyidae s.str.: Phyllocoptinae: *Leipothrix nagyi*
*ve*	drop-shaped (Figure 1H)	Phytoptidae s.str.: in some *Propilus* (e.g., *Propilus bactris*)
*sc*, *ve*	with one or two basal swellings (Figure 1I,J)	Phytoptidae s.str.: *Retracrus* *

**Table 3:** Characteristics of mitochondrial genomes of four *Leipothrix* spp. Notations: sp1—*Leipothrix aegopodii*, sp2—*Leipothrix* sp. A, sp3—*L. knautiae*, sp4—*L. convallariae*, J—positive chain of mitochondrial DNA, N—negative chain of mitochondrial DNA. In the first column, the codons are given in brackets after each corresponding tRNA. Numbers in brackets indicate the amounts of overlapping nucleotides between adjoining genes (the minus indicates genes located on the negative chain).

Gene	Strand	Position and Intergenic Nucleotides				Size			
		sp1	sp2	sp3	sp4	sp1	sp2	sp3	sp4
*COX1*	J	1–1578; 13548–13550	1–1578; 13610–13615	1–1578; 13694–13702	1–1587(1)	1581	1584	1587	1587
*COX2*	J	1590–2249	1580–2239	1590–2246	1587–2240	660	660	657	654
trn*D* (cag)	J	2250–2302	2240–2296	2260–2318	2241–2298	53	57	59	58
*ATP8*	J	2303–2461 (1)	2297–2452 (1)	2319–2474 (1)	2299–2454 (1)	159	155	156	156
*ATP6*	J	2461–3108 (1)	2452–3096 (1)	2474–3121 (1)	2454–3101	648	644	648	648
*COX3*	J	3108–3908	3096–3893 (1)	3121–3912	3105–3899	801	798	792	795
trn*G* (gga)	J	3909–3973	3893–3953 (2)	3913–3963	3912–3967	65	51	51	56
*NAD3*	J	3974–4306	3952–4293	3964–4294	4005–4301	333	342	331	297
trn*A* (gca)	J	4307–4353	4294–4333	4296–4346	4302–4351 (2)	47	42	51	50
trn*R* (cga)	J	4354–4394	4334–4383 (2)	4347–4390	4350–4387 (1)	41	50	44	38
trn*N* (aac)	J	4395–4449 (5)	4382–4438	4391–4446	4387–4442 (5)	55	56	56	56
trn*S* (aga)	J	4445–4496 (3)	4439–4480	4447–4489	4438–4486	52	43	43	49
trn*E* (gaa)	J	4498–4552 (1)	4481–4540	4499–4553	4490–4543	55	60	55	54
trn*I* (atc)	J	4552–4616 (1)	4541–4595	4563–4613	4552–4611	64	55	51	60
trn*F* (ttc)	N	4616–4687 (−2)	4596–4660	4635–4694	4612–4679	72	65	60	68
*NAD5*	N	4686–6206	4661–6184	4696–6216	4679–6194	1521	1524	1521	1516
trn*H* (cac)	N	6207–6261	6185–6241	6217–6271	6195–6252	55	57	55	58
*NAD4*	N	6264–7496	6242–7477 (−1)	6274–7515 (−4)	6255–7487 (−1)	1233	1236	1242	1233
*NAD4L*	N	7498–7776	7477–7749	7512–7784	7487–7759	279	273	273	273
trn*P* (cca)	N	7777–7829	7750–7803 (−1)	7785–7838	7760–7813 (−1)	53	53	54	54
*NAD6*	J	7830–8279	7803–8252	7838–8290	7813–8262	450	450	453	450
trn*T* (aca)	J	8280–8328	8253–8299	8289–8336 (2)	8263–8312	49	47	48	50
*CYTB*	J	8329–9426 (1)	8300–9397	8337–9434	8311–9408 (2)	1097	1098	1098	1098
trn*S* (tca)	J	9425–9474	9398–9443	9433–9482	9407–9456 (1)	50	46	50	50
*NAD1*	N	9473–10369	9444–10340	9481–10377 (−2)	9456–10352 (−1)	897	897	897	897
trn*L* (cta)	N	10370–10430	10341–10403	10378–10438	10352–10414 (−2)	61	63	61	63
trn*W* (agt)	J	10439–10503	10403–10472	10439–10507	10413–10467	65	70	69	55
trn*V* (gta)	J	10504–10556	10473–10527	10506–10560	10482–10534	53	55	55	53
rrnS	J	10557–11277	10528–11247	10561–11293	10535–11275	721	720	733	741
rrnL	J	11278–12238	11248–12213	11294–12252	11276–12248	961	966	960	973
trn*Y* (tac)	J	12243–12296 (2)	12215–12261	12255–12308 (3)	12249–12302	54	54	54	54
trn*L* (tta)	J	12294–12354	12262–12322	12306–12366	12303–12350	61	61	61	48
*CR*	J	12355–12410	12323–12440	12367–12551	12351–12389	56	118	185	39
*NAD2*	J	12411–13337 (6)	12441–13394 (7)	12552–13481	12390–13265	927	954	930	876
trn*Q* (caa)	N	13332–13385	13388–13443	13486–13531	13260–13314	54	56	46	55
trn*C* (tgc)	N	13386–13428	13444–13484	13534–13576 (–2)	13319–13363	43	41	43	45
trn*M* (atg)	J	13429–13484 (1)	13485–13541	13575–13631	13366–13421 (1)	56	57	57	56
trn*K* (aaa)	J	13484–13547	13542–13609	13632–13694 (1)	13421–13483	64	68	63	63

## Data Availability

All new DNA sequences obtained in this study have been deposited in the National Center for Biotechnology Information (NCBI) GenBank database (https://www.ncbi.nlm.nih.gov/genbank) (accessed on 3 May 2023).
